# Experiences and perspectives of Ayurvedic practitioners on non-communicable disease management in the UK: A qualitative study

**DOI:** 10.1371/journal.pone.0360754

**Published:** 2026-10-12

**Authors:** Patricia Egwumba, Manpreet Bains, Laura Nellums, Kaushik Chattopadhyay

**Affiliations:** 1 Lifespan and Population Health, School of Medicine, University of Nottingham, Nottingham, United Kingdom; 2 The Nottingham Centre for Evidence-Based Healthcare: a JBI Centre of Excellence, Nottingham, United Kingdom; 3 College of Population Health, Health Sciences Centre, University of New Mexico, New Mexico, United States of America; North-Eastern Hill University, INDIA

## Abstract

**Introduction:**

Non-communicable diseases (NCDs) are a major public health challenge in the United Kingdom (UK), placing strain on the National Health Service (NHS). While Western medicine dominates NCD care, the use of complementary and alternative medicine (CAM), such as Ayurveda, is increasing. The objective of this study was to explore the experiences and perspectives of Ayurvedic practitioners in the UK regarding the use of Ayurveda for managing NCDs.

**Methods:**

Seventeen UK-based Ayurvedic practitioners participated in semi-structured qualitative interviews that were audio-recorded, transcribed verbatim, and analysed thematically.

**Findings:**

Three key themes were identified. First, Ayurvedic practitioners viewed their role as complementary to Western medicine, providing holistic, individualised, and patient-centred care to support NCD management. Second, they faced multiple barriers in the UK, including structural challenges (limited integration, poor institutional support, and restricted patient access) and financial constraints (high treatment costs, lack of NHS coverage, and import regulations). Finally, professional associations played a vital role in providing legal and regulatory guidance, promoting continuous professional development, and supporting research to strengthen evidence and legitimacy.

**Conclusion:**

Although Ayurvedic practitioners face considerable challenges in the UK, they play a unique role in negotiating legitimacy, professional recognition, and the epistemological challenges of practising within a Western-dominated healthcare system. Professional associations help legitimise and advance their practice, supporting culturally congruent, patient-centred care that complements conventional NCD management. Despite structural barriers, Ayurvedic practitioners have maintained their presence and have navigated a complex, structurally Western-centric healthcare landscape, offering insights into integrative approaches to NCD care.

## 1. Introduction

Non-communicable diseases (NCDs) are conditions that are not transmitted from person to person; however, they typically have a long duration and slow progression [1,2]. In the United Kingdom (UK), NCDs pose a significant public health and economic challenge, accounting for 89% of years lived with disability (YLDs), 81% of disability-adjusted life years (DALYs), and 76% of all deaths [3]. The increasing prevalence of NCDs places a growing burden on the National Health Service (NHS) and reduces workforce productivity [4–6].

In addition to conventional care, the use of complementary and alternative medicine (CAM) has been steadily increasing in the UK [7–11]. Recent surveys have reported that approximately 12% of individuals consult a CAM practitioner annually, compared with 10% in earlier surveys [12]. Overall, it has been estimated that approximately 44% of individuals in the UK have used CAM in their lifetime, with most access occurring through the private sector [12,13]. Importantly, CAM use has also been reported among NHS priority groups commonly affected by NCDs, including individuals with cancer (11%), those managing diabetes or coronary heart disease (5%), and those with mental health concerns (9%) [13]. Most of this occurs outside the NHS, with patients purchasing over-the-counter products or consulting private practitioners [12,13]. CAM is frequently used as a complement to conventional care to treat conditions for which Western medical treatment options are limited and to enhance the overall state of health [12,14]. Motivations for CAM use include dissatisfaction with conventional care, a desire to avoid pharmaceutical side effects, and a preference for natural or traditional healing approaches [12,14]. Together, these trends suggest that CAM may play a growing role in the management of NCDs in the UK.

Ayurveda, a traditional medical system that originated in the Indian subcontinent over 5000 years ago, is a form of CAM that emphasises lifestyle and dietary modifications [15,16]. It also uses Ayurvedic detoxifying and purifying therapies (e.g., Panchakarma) and Ayurvedic medicines (containing plant-, animal-, or mineral-origin ingredients–single or in combination) [15,16]. Despite being classified as an ‘alternative’ medical system in the UK [17,18], Ayurveda is often used by people with NCDs, highlighting the need to better understand its potential contribution to NCD management [10,17,19]. However, despite evidence of CAM use among individuals with NCDs, there are currently no national data on Ayurveda use specifically among people with NCDs in the UK, underscoring a gap in understanding its role and reach within this context.

Existing qualitative studies have explored aspects of the adaptation of Ayurveda in the UK, including its spiritual framing, and Ayurvedic practitioners’ self-exploration of their practice and training, but have not specifically focused on NCD management [9,20]. Hence, there remains a gap in understanding how Ayurvedic practice operates within the UK’s plural healthcare landscape [20]. The objective of this study was to explore Ayurvedic practitioners’ experiences and perspectives on managing NCDs in the UK.

## 2. Methods

### 2.1. Study design, ethics, and reporting

This qualitative study was underpinned by the constructivist philosophical paradigm and was conducted in the UK using a descriptive exploratory approach [21,22]. The study was approved by the Research Ethics Committee, Faculty of Medicine and Health Sciences, University of Nottingham (approval number: FMHS 298-0523). The study was reported according to the Consolidated Criteria for Reporting Qualitative Research (COREQ‐32) guideline [23].

### 2.2. Participant recruitment and sampling

Ayurvedic practitioners were eligible to participate if they possessed at least a diploma or degree in Ayurveda, had been practising in the UK, and had at least one year of experience treating patients with NCDs. Purposive sampling was employed to ensure that the participants had relevant experience in the management of NCDs [21], allowing the study to capture in-depth insights from practitioners with direct exposure to NCD care and ensuring the data would be rich, relevant, and informative for the research aim [21,22].

Potential participants were contacted through two Ayurvedic associations and groups in the UK, namely, the Ayurveda Professional Association (APA) and the All-Party Parliamentary Group on Indian Traditional Sciences (APPG); both associations circulated the study poster containing the lead researcher’s (PE) contact details among their members. A total of 23 Ayurvedic practitioners initially contacted the lead researcher. Of these, 20 remained in contact following the initial communication, while three declined to participate, citing unavailability and timing constraints. The remaining 20 practitioners were provided with a participant information sheet and consent form via email. The lead researcher responded to any questions raised. Interested practitioners were given at least 24 hours to consider participation before interviews were scheduled. Three practitioners did not respond to subsequent follow-up emails. Interviews were therefore conducted with the eligible practitioners who confirmed their willingness to participate. Written informed consent was obtained from all participants prior to data collection. Recruitment for this study began on July 13, 2023, and was completed by March 30, 2024.

In qualitative research using semi-structured interviews, sample size is guided by analytic adequacy rather than a predetermined numerical target [21,22]. Traditionally, this has been conceptualised through thematic saturation, defined as the point at which further data collection yields little or no new information relevant to the research question [21,22]. In this study, sample adequacy was considered in relation to both thematic saturation and the concept of information power, which emphasises the relevance, richness, and experiential specificity of participant data and proposes that samples containing richer and more relevant information require fewer participants [21,22].

Sample adequacy and data saturation were monitored iteratively throughout recruitment. Following each set of interviews, emerging patterns were reviewed to assess whether the sample was sufficient to address the research question and whether new issues continued to arise. By the 14th interview, no substantively new themes were identified, suggesting that thematic saturation had been reached. Three further interviews were conducted to confirm stability of the findings and ensure no additional areas of conceptual relevance emerged. Recruitment was subsequently discontinued, supporting analytic adequacy and resulting in a final sample of 17 practitioners. This sample size was considered sufficient to address the study’s objective and generate a rich, in-depth understanding of Ayurvedic practitioners’ experiences of NCD management in the UK.

### 2.3. Interview guide and data collection

An interview guide was developed by the research team based on the study’s objective and existing relevant literature. Probing and open-ended questions were incorporated into the guide to encourage Ayurvedic practitioners to openly express their opinions. The guide was piloted with two Ayurvedic practitioners, whose data were included in the study because no revisions were required, and their responses were analytically comparable to subsequent interviews. The lead researcher conducted all interviews in English, using the guide and Microsoft Teams. All interviews were audio-recorded with consent. The process and purpose of the study were reiterated to participants before each interview.

### 2.4. Data analysis

Two interview recordings were transcribed verbatim by the lead researcher, while the remaining recordings were transcribed by a University of Nottingham-approved external transcription company. The lead researcher checked the transcripts for accuracy and anonymised any identifiable information. NVivo 14.0 was used to facilitate data management and interpretation [24].

Data were analysed thematically using Braun and Clarke’s six-phase process [25]. To facilitate familiarisation, the lead researcher read and re-read the transcripts. Transcripts were then coded line-by-line inductively, with codes assigned to meaningful segments of data. To enhance analytic rigour, MB independently double-coded seven of the 17 transcripts, representing a substantial proportion of the dataset and enabling comparison and discussion of coding and thematic interpretations. This process served as a form of investigator triangulation rather than a means of achieving coding consensus, allowing multiple analytic perspectives to challenge, refine, and strengthen interpretations and analytic decisions [21,22]. Coding decisions were discussed between PE and MB, leading to refinement of the coding framework.

These processes enhanced the trustworthiness of the analysis by supporting credibility, dependability, and confirmability through investigator triangulation, reflexive engagement, ongoing discussion and refinement of analytic interpretations, and systematic documentation of analytic decisions. Participant validation was undertaken with five participants to clarify specific statements, particularly those relating to Ayurvedic concepts and terminology, thereby ensuring the accurate interpretation and representation of their perspectives. This process confirmed the researcher’s understanding of these concepts rather than validating the thematic analysis. Interpretations remained grounded in participants’ accounts and verbatim quotations.

In the subsequent phases, the lead researcher reviewed, merged, and organised the codes into subthemes, with ongoing input from the other two researchers (MB and KC). These subthemes were synthesised into overarching themes, which were then reviewed, refined, and analytically defined and named, resulting in a final set of themes.

### 2.5. Reflexivity

The research team acknowledged that their professional backgrounds could influence data collection and analysis. As a trained Western medicine practitioner, the lead researcher recognised that limited exposure to Ayurvedic principles and practices could shape interpretation of the data. To minimise potential bias, reflexive journaling and bracketing were undertaken, prioritising participants’ perspectives to ensure the analysis remained grounded in the data.

The research team also recognised that KC, although trained as an Ayurvedic practitioner, is a senior evidence-based healthcare researcher with extensive experience in critically interpreting research findings. This background provided valuable contextual knowledge while maintaining a strong focus on evidence-based analysis grounded in participants’ accounts and study findings. MB, a senior qualitative researcher without a clinical background in Ayurveda, contributed an independent methodological perspective that supported critical reflection and refinement of theme development.

## 3. Results

### 3.1. Participant characteristics

We interviewed 17 UK-based Ayurvedic practitioners. The average duration of the interviews was 85 minutes (range: 50–120 minutes). The characteristics of participating Ayurvedic practitioners are presented in Table 1. Most  participants held a Bachelor of Ayurvedic Medicine and Surgery (BAMS) or higher qualification and had been practising in the UK for over a decade. While clinic schedules varied, nearly half practised fewer than three days per week, and most saw fewer than 20 patients weekly. Participants reported having treated various types of NCDs, mostly belonging to the musculoskeletal, respiratory, digestive, skin, and endocrine systems. Arthritis, asthma, irritable bowel syndrome, eczema, diabetes, and cancer were among the most frequently treated conditions in these categories.

**Table 1:** Characteristics of participating Ayurvedic practitioners.

Characteristic	N (%)
Age (years), Mean ± SD	55 ± 13
Sex	Female: 10 (58.8%)
	Male: 7 (41.2%)
Ethnicity	White British: 3 (17.6%)
	British Asian: 14 (82.4%)
Highest Ayurveda qualification*	Diploma: 3 (17.6%)
	BAMS: 9 (52.9%)
	MD: 4 (23.5%)
	PhD: 1 (5.9%)
Practising Ayurveda in the UK	<10 years: 3 (17.6%)
	10–20 years: 5 (29.4%)
	>20 years: 9 (52.9%)
Clinic location in the UK	England: 15 (88.2%)
	Northern Ireland: 2 (11.8%)
Clinic days/week	<3 days: 8 (47.1%)
	3–4 days: 5 (29.4%)
	5–7 days: 4 (23.5%)
Average number of patients with NCDs seen/week	<10 patients: 8 (47.1%)
	10–20 patients: 4 (23.5%)
	>20 patients: 5 (29.4%)

*Diploma: 2 years; BAMS: 4.5 years plus 1-year internship; MD: 3 years of clinical training and research; PhD: 3–5 years depending on institution. All PhD and MD holders also had a BAMS. BAMS and higher degrees are medical qualifications in South Asia, conferring the title ‘Dr.’, which diploma holders do not receive.

### 3.2. Qualitative themes

Thematic analysis of the qualitative data identified three main themes, each comprising several sub-themes, which together illustrate the experiences and perspectives of Ayurvedic practitioners in the UK regarding NCD management. S1 Table summarises these themes and subthemes, highlighting practitioners’ roles in NCD management, barriers to practice, and the support provided by professional associations. Illustrative quotes are included to exemplify participants’ perspectives and experiences for each theme. Participant quotes were anonymised and assigned the label “AP” (Ayurvedic Practitioner). Quotes are presented verbatim unless indicated otherwise by an ellipsis (…) to reflect minor omissions made for clarity and readability.

#### 3.2.1. Role of Ayurvedic practitioners in NCD management.

This theme describes how Ayurvedic practitioners co-managed patients with Western medical practitioners. It captured the dual perspectives of Ayurvedic practitioners on NCD management: It showed how they applied Ayurvedic principles to understand and treat these conditions while also recognising and navigating the Western medical framework.

##### 3.2.1.1. Negotiating the role of Ayurveda alongside Western medicine:

In a medical system dominated by Western medicine, most practitioners trained solely in Ayurveda perceived their role in NCD management as complementary rather than a replacement for Western treatment. They emphasised collaboration and noted that integrating Ayurveda with Western medical treatments could benefit NCD management.

*“We need to work hand in hand, not criticise each other, as there are areas in Western medicine that Ayurveda may be helpful for, such as NCD management.” (AP15)*

Many Ayurvedic practitioners shared their experiences of co-managing patients with NCDs with Western medical practitioners, for instance, in cancer treatment. Most Ayurvedic practitioners acknowledged the critical role of surgery and chemotherapy in cancer treatment; however, they also mentioned how they integrated Ayurvedic therapies to support patient recovery and prevent recurrence.

*“The Western medical system excels in areas like surgery, which is crucial. When a patient with breast cancer came to me, I advised her, ‘…you must have the surgery.’ She underwent surgery and chemotherapy, and we used Ayurveda to prevent recurrence.” (AP5)*

Two Ayurvedic practitioners who were also qualified Western medicine practitioners demonstrated the complementary role of Ayurveda in the management of NCDs. These individuals combined Ayurveda and Western medicine by leveraging their knowledge of both systems; they were able to provide holistic treatment. They used Western medicine for life-threatening management while introducing Ayurveda for long-term, milder, safer options for symptom control.

*“As a GP or surgeon, I would usually prescribe ibuprofen, voltarol, or naproxen, anti-inflammatory drugs that can have harsh adverse effects, such as ulcers. The role of Ayurveda is to provide a much milder and safer alternative with natural herbal preparations. I integrate it into my practice.” (AP6)*

While most Ayurvedic practitioners emphasised the complementary role of Ayurveda alongside Western medicine, a few practitioners trained solely in Ayurveda took a more oppositional stance. They emphasised that Ayurveda should be used as a complete, stand-alone alternative for managing NCDs, particularly in cases where Western medicine offered limited relief or when patients preferred non-pharmaceutical options. These Ayurvedic practitioners shared their clinical experiences in which people with NCDs who initially relied on Western medication for temporary relief were able to transition to Ayurveda for long-term management and sustained well-being.

*“In India, Ayurveda is mainstream, whereas here it is treated as complementary. Sometimes people just don’t get much improvement with modern medicine, and there are lots of people who don’t want to take modern medicine too, so they look for natural methods like Ayurveda.” (AP3)*

To clarify how practitioners’ professional background influenced their perspectives on the role of Ayurveda in NCD management, Table 2 presents the background, stance, and illustrative quotes of the study.

**Table 2:** Mapping practitioner professional background to perspectives on the role of Ayurveda in NCD management.

Practitioner Background	Perspective on Role of Ayurveda	Illustrative Quotes
Practitioners trained solely in Ayurveda (majority: 15 practitioners)	**Complementary** (13 practitioners): These practitioners view Ayurveda as complementary to Western medicine. **Standalone** (2 practitioners): These practitioners view Ayurveda as a standalone treatment.	**Complementary:** *“We need to work hand in hand, not criticise each other, as there are areas in Western medicine that Ayurveda may be helpful for, such as NCD management.” (AP15)* *“The Western medical system excels in areas like surgery, which is crucial. When a patient with breast cancer came to me, I advised her, ‘… you must have the surgery.’ She underwent surgery and chemotherapy, and we used Ayurveda to prevent recurrence.” (AP5)* **Standalone:** *“I think Ayurveda is one of the natural systems of health and wellness for prevention and health promotion. It stands as a mainstream alternative, with many people turning to such systems at some point in their lives.” (AP8)* *“In India, Ayurveda is mainstream, whereas here it is treated as complementary. Sometimes people just don’t get much improvement with modern medicine, and there are lots of people who don’t want to take modern medicine too, so they look for natural methods like Ayurveda.” (AP3)*
Dual-trained practitioners (2 practitioners)	These practitioners combine Ayurveda and Western medicine.	*“As a GP or surgeon, I would usually prescribe ibuprofen, voltarol, or naproxen—anti-inflammatory drugs that can have harsh adverse effects, such as ulcers. The role of Ayurveda is to provide a much milder and safer alternative with natural herbal preparations. I integrate it into my practice.” (AP6)* *“I don’t say Ayurveda has a cure for all conditions; it has its limitations. At least for certain conditions where it does help, it can be complementary.” (AP12)*

##### 3.2.1.2. Patient-centred approach and empowerment:

Ayurvedic practitioners emphasised their personalised approach to NCD management, highlighting how it differs from the one-size-fits-all approach of Western medicine. Ayurvedic practitioners revealed that Ayurveda’s holistic approach considers the entire individual, including lifestyle, diet, emotional well-being, and underlying imbalances. They explained that all these factors influence an individual’s health and well-being.

*“In Ayurveda, we primarily see each person as an individual. It is a patient-centred approach rather than a disease-centred one … Even if two people had the same diagnosis, they would not necessarily receive the same treatment.” (AP3)*

Beyond prescribing herbal medicines, many practitioners saw their role as empowering patients to take an active part in their health. This often requires adjustments, such as lifestyle and dietary changes, with practitioners guiding patients through these modifications. Over time, they reported that many patients became more aware of how their daily habits influenced overall well-being, particularly in relation to NCDs. Ayurvedic practitioners expressed deep satisfaction in reporting that some patients appeared to take greater control of their health and make meaningful lifestyle changes.

*“A patient’s blood pressure medication was doubled without lifestyle advice. I prescribed herbs and recommended walking and diet changes—now she is off the medication.” (AP11)*

At the same time, practitioners acknowledged that patient expectations could pose challenges. Many patients sought Ayurveda after trying conventional treatments, often presenting at a later stage of their condition and expecting immediate relief, reflecting Western medical paradigms that prioritise rapid results. Practitioners emphasised that guiding patients to shift their mindset from a disease-focused model to one centred on long-term prevention and holistic well-being is essential, as Ayurveda requires patience and commitment.

*“I notice that people are very used to getting medication with quick effects … so they may have the same expectations with Ayurveda.” (AP2)*

#### 3.2.2. Barriers to Ayurvedic practice in the UK.

This theme explores the key barriers that limited Ayurvedic practitioners’ ability to deliver and sustain care for people living with NCDs in the UK. Participants described structural and financial challenges, including the lack of statutory regulation, limited integration with Western medicine, resource constraints, and the high cost of providing and accessing Ayurvedic care.

##### 3.2.2.1. Structural barriers:

These are the barriers created by the health system structure that prevent Ayurvedic practitioners from making their services accessible to individuals with NCDs. Such barriers are entrenched in policy, regulation, and institutional frameworks and have impacted the ability of Ayurvedic practitioners to work with Western medical practitioners to provide holistic care to patients with NCDs.

*3.2.2.1.1. Professional independence, accountability, and standardisation gaps.* Ayurvedic practitioners emphasised that both institutional backing and formal recognition are critical for effective NCD management, but they are largely absent in the UK. In India, Nepal, and other countries in the Indian subcontinent, practitioners work across the full spectrum of healthcare services, including primary health centres, community clinics, outpatient departments, and inpatient hospital settings, where responsibility for patient outcomes is shared collaboratively. In contrast, most UK practitioners work independently, without institutional support or statutory regulation, leaving them solely accountable for patient outcomes and professional credibility.

*“As an Ayurvedic practitioner, I must be cautious about what I prescribe. If I prescribe something outside the pharmacopoeia … I will be responsible for that. Nobody is there to support me in case there is a problem.” (AP5)*

Moreover, practitioners also reported that qualifications from reputable institutions abroad are not recognised in the UK, and the development of standardised training, accreditation, and practice guidelines is hindered by the absence of statutory regulation. Without regulation, unqualified individuals could present themselves as practitioners, raising concerns about patient safety and public trust.

*“We are not a regulated profession; it would be good if it was regulated because then only people who are fully qualified can practice.” (AP3)*

The lack of formal regulation and standardised practice guidance meant that UK practitioners often lacked a clear framework for delivering care.

*“There isn’t really a blueprint for how people should deliver or do their practice… There is no standardised approach to how we practice.” (AP2)*

Despite these challenges, practitioners expressed a strong commitment to providing high-quality treatment for people with NCDs, navigating these structural gaps with caution and professionalism.

*3.2.2.1.2. Resource limitations and adjustments in practice.* Ayurvedic practitioners highlighted the challenges of limited access in the UK to essential resources needed to manage patients with NCDs, such as fresh herbs, potent mineral formulations, and specialised facilities for intensive therapies such as Panchakarma. In contrast to India, where these resources are readily available, UK Ayurvedic practitioners had to creatively adapt their approaches while seeking to maintain treatment effectiveness. Practitioners described innovative strategies, such as using high-quality dried herbs, modifying formulations, and tailoring NCD treatments to the available resources.

*“In this country, fresh herbs for managing NCDs are not so readily available; if they are, it’s too expensive, and there are no Panchakarma facilities … so we must make do with what we have.” (AP17)*

*3.2.2.1.3. Barriers to patient referrals and integration with Western medicine.* A major challenge for Ayurvedic practitioners in the UK was the general lack of willingness among Western medical practitioners to refer patients with long-standing NCDs. Ayurvedic practitioners noted that most of this reluctance stemmed from scepticism about the safety of Ayurvedic medicine in relation to NCD management or limited awareness of the potential benefits of using Ayurveda. Ayurvedic practitioners reported that the reluctance of Western medical practitioners to refer patients to Ayurveda restricted patients’ access to Ayurvedic care, which they believed could benefit individuals with various NCDs as part of an integrated approach to healthcare.

*“The access to patients with NCDs is very limited here … it is difficult to get referrals from GPs or other Western medical specialists for Ayurveda, which requires a policy change.” (AP1)*

Ayurvedic practitioners called for a policy review to include Ayurveda in mainstream healthcare and educational programmes to increase Western medical practitioners’ awareness of Ayurvedic approaches to NCD management.

##### 3.2.2.2. Financial barriers:

Access to Ayurvedic services was limited by financial barriers, mainly due to the lack of NHS coverage and the cost of importing Ayurvedic medicines. Participants felt that this made Ayurveda less affordable and accessible, particularly for individuals with lower incomes.

*3.2.2.2.1. Financial and regulatory challenges affecting Ayurveda access.* Ayurvedic practitioners reported substantial financial and market challenges, which limited patient access to Ayurveda and their ability to practice in the UK. Lack of NHS coverage for treatment is a major challenge. Individuals with NCDs who wished to use Ayurveda had to pay out of pocket, as it was not covered under the NHS. This made Ayurveda appear as a premium service, limiting accessibility to those who could afford private care. Practitioners reported that most patients with NCDs in the UK had obtained a medical exemption certificate that granted them free access to NHS prescriptions, creating competitive environments for alternative medicines.

*“In the NHS, NCD treatment is a bit free, whereas in other countries it’s not the same; with having the NHS free, you are competing with that for the patients.” (AP1)*

Financial barriers also influenced the continuity of care. Ayurvedic practitioners reported that some patients discontinued treatment because of the cost, which they perceived as limiting the potential effectiveness of Ayurvedic treatment. However, many practitioners have also modified their practice to provide quality affordable care.

*“Since Ayurveda is not covered by the NHS, you must pay for it privately; thus, many of the clients discontinue treatment because of the cost.” (AP4)*

Financial barriers not only restricted patient access but also threatened the sustainability of Ayurvedic practice in the UK. Several practitioners reported struggling to make a living solely from Ayurveda, forcing them to pursue additional jobs.

*“Making a living from it is a big challenge because it’s not popular. I had to conduct other studies in psychology and do another job.” (AP5)*

In addition to the high treatment costs, practitioners had to bear high import costs and stringent regulatory requirements for Ayurvedic medicines. Medicines had to be approved by the Medicines and Healthcare Products Regulatory Agency (MHRA), requiring long and costly testing protocols. This deterred many reputable manufacturers from South Asia from investing in the UK market, leaving only a limited number of approved suppliers and driving up procurement costs.

*“The MHRA restricts the import of Ayurvedic medicines. They must be licenced here and submitted for research. Indian companies are not prepared to invest in the UK … because the returns are not there.” (AP6)*

Practitioners emphasised the importance of ethical sourcing and rigorous quality control, including testing for contaminants such as heavy metals. While these measures are essential for patient safety, they significantly added to financial pressures.

*“Regarding testing, ideally, you want to be testing for heavy metals and pollutants in herbs. In the UK, it’s a lot more expensive to do the testing … ideally every batch that comes from India should be tested, because they have different standards in India than here” (AP3)*

Ultimately, the combined burdens of high treatment costs, lack of NHS coverage, licensing and testing requirements, and expensive imports constrained patient access to Ayurveda while straining the practice’s sustainability in a financially unsupportive environment.

#### 3.2.3. Benefits of professional associations and networks.

Professional associations and networks have played a significant role in facilitating the practice of Ayurveda in the UK. Ayurvedic practitioners stressed their role in regulating, maintaining, and expanding their professional work.

##### 3.2.3.1. Professional guidance on legal and regulatory frameworks:

According to Ayurvedic practitioners, professional associations were important to help navigate the complex legal and regulatory terrain of the UK. These associations provided legal and ethical guidance on how to comply with UK health regulations. This regulatory support was vital in the context of managing NCDs because the continuity of care and patient safety were of great value. By educating practitioners on what claims they could make and what safe practices were for treating NCDs, associations could better equip Ayurvedic practitioners to provide Ayurvedic treatment for NCDs with more confidence and accountability. Membership included access to practitioner insurance for those without standard medical credentials, which was particularly beneficial due to its support in allowing practitioners to provide long-term, structured care for patients with NCDs.

*“Associations like the British Association of Accredited Ayurvedic Practitioners, or APA, once you are a member, you can get insurance from holistic insurance … that facilitates the practice of Ayurveda in this country.” (AP6)*

##### 3.2.3.2. Continuous Professional Development (CPD) support:

Ayurvedic practitioners practising in the UK emphasised the vital role of professional associations and networks in supporting their ongoing clinical education, maintaining high standards of care, and advancing their professional development. These organisations were seen as central to promoting best practices, sharing collaborative knowledge, and enhancing the credibility of Ayurveda in managing NCDs. Ayurvedic practitioners indicated that CPD events organised by associations were particularly important for staying informed about new treatment methods for NCDs and integrated care models. These interventions also represented opportunities for joint discussion, peer learning, and mentoring and contributed to the development of a ‘culture of continuous improvement’ in clinical skills associated with NCD treatment.

*“We have Ayurvedic professional associations, and they do CPD courses and things we can join to keep our practice up to date.”* *(AP5)*

##### 3.2.3.3. Evidence generation and research support:

Ayurvedic practitioners highlighted the role of professional associations in promoting the scientific validation of Ayurveda in the UK. Although evidence on Ayurveda’s use in the management of NCDs was seen as emerging in the UK, the participants emphasised the demand for stronger and peer-reviewed evidence that could support its integration into conventional healthcare systems. They emphasised that professional associations were well-positioned to facilitate and promote collaborative research with Western medical institutions, particularly around NCD care. By endorsing such initiatives, associations have contributed to enhancing the acceptability of Ayurvedic management of NCDs and opened opportunities for its recognition.

*“There is some research happening now … Integration requires much research, but without collaboration and research, we cannot bring it to policy.” (AP1)*

## 4. Discussion

This study provides novel insights into the experiences and perspectives of Ayurvedic practitioners in the UK regarding the use of Ayurveda in the management of NCDs. Practitioners described applying Ayurvedic principles to support long-term management, while navigating regulatory, professional, and financial challenges within a Western-dominated healthcare system. Participants consistently highlighted the role of professional associations in sustaining clinical practice, education, and research. These findings align with previous research on Ayurveda and CAM, particularly in relation to tensions between integration with Western medicine and professional autonomy [9,20,26–31].

In relation to patient engagement and clinical practice, practitioners emphasised holistic and patient-centred approaches to care. However, patient adherence emerged as a key challenge. Participants reported that some patients expected rapid symptom relief and found the longer-term commitment required for Ayurvedic care difficult to maintain [32–34]. Treatments were often perceived as “slow-acting” compared to conventional medicine [17,19,35,36]. These findings suggest that adherence is influenced not only by perceived effectiveness but also by patient expectations and broader social factors [37,38].

At a structural level, practitioners identified several barriers, including a lack of formal recognition, limited integration within the NHS, and minimal institutional support. These constraints restricted collaboration with Western medical practitioners, limited referral pathways, and often required practitioners to work independently. These findings align with existing literature describing challenges related to regulation, accreditation, professional recognition, and the integration of Ayurveda and other traditional or complementary practices [9,20,26–30] and reflect broader discussions of the marginalisation of non-Western medical systems [39]. Cross-cultural observations have similarly highlighted differences in how Ayurveda is positioned across healthcare contexts, with Ayurveda functioning as an established clinical system in India while being more commonly associated with diet, lifestyle, and complementary care in Western settings [40].

Participants also emphasised the financial implications of these structural constraints. Reliance on out-of-pocket payments was reported to contribute to treatment discontinuation and fragmented care, particularly among patients with limited financial resources. Sustained Ayurvedic care was therefore difficult to maintain in some cases. Practitioners also reported challenges in sustaining Ayurveda as a sole source of income, leading some to reduce clinical hours or combine practice with other employment.

In response to these challenges, professional associations were described as playing a central role in sustaining Ayurvedic practice. Participants highlighted their contributions to legal and ethical guidance, insurance provision, and self-regulation within an ambiguous regulatory environment. Associations were also described as supporting CPD and maintaining professional standards. These findings resonate with wider research on professional positioning and legitimacy among CAM practitioners [29,39].

Professional associations were further perceived to prioritise scientific validation as a means of strengthening Ayurveda’s legitimacy. Participants emphasised the importance of rigorous, peer-reviewed research and expressed optimism about collaborative initiatives with Western medical institutions. These perspectives are consistent with wider discussions concerning evidence generation and the methodological and epistemological challenges associated with integrating Ayurveda and other complementary approaches into conventional healthcare [41,42].

Taken together, these findings highlight how Ayurvedic practitioners negotiate legitimacy and professional sustainability within a constrained healthcare context. They extend CAM integration debates by foregrounding practitioner perspectives within a less institutionally recognised tradition [43,44]. These findings resonate with wider literature describing challenges of professionalisation, legitimacy, and institutional recognition within CAM and traditional medicine [29,39,43–45]. These dynamics are shaped by broader structural and epistemic factors [42,46–48] and suggest that legitimacy is influenced not only by evidence of clinical effectiveness but also by professional identity, boundary work, and positioning within healthcare systems [38,39,49,50].

Practitioners’ accounts also point to implications for education and health policy. Participants suggested that incorporating Ayurveda into health and medical education, for example, through postgraduate or certificate-level programmes, could support safer and more evidence-informed integrative care. This aligns with wider literature identifying gaps in education about complementary and integrative approaches among healthcare professionals [51]. Such educational approaches may facilitate greater interdisciplinary understanding and collaboration between Western medical and traditional healthcare practitioners [46,51].

### 4.1. Strengths and limitations of the study

This study is the first qualitative investigation of Ayurvedic practitioners’ experiences and perspectives in the UK in relation to NCD management, contributing to research on medical pluralism and traditional medicine in contemporary healthcare settings. Although conducted in the UK, the findings may be relevant to other multicultural contexts. The use of semi-structured interviews and thematic analysis enabled an in-depth exploration of practitioner perspectives [21]. While the interpretation was informed by literature from medical sociology and anthropology [37,38,52], the analysis remained grounded in the data [21].

One limitation is that interviews were conducted online rather than face-to-face, which may have altered opportunities to observe non-verbal cues; however, videoconferencing also facilitated participation and flexibility [53]. Nearly half of the participants reported seeing fewer than ten patients with NCDs per week, which may limit the transferability of findings to higher-volume practice settings [21,22]. As these figures referred specifically to NCD cases, they may not reflect overall clinical workload.

The use of purposive sampling and recruitment through professional associations may have introduced selection bias, as participants were likely to be more professionally engaged [21,22]. In addition, the sample was relatively homogeneous, limiting the diversity of perspectives and reducing transferability.

As with all practitioner-based qualitative research, participants’ accounts may reflect aspects of professional positioning; the authors considered this analytically. The study did not specifically explore practitioners’ condition-specific expertise or the systematic documentation and sharing of individual clinical observations. Future research could examine these aspects in greater depth, including how practice-based knowledge is documented, shared, and incorporated into professional learning. This study did not specifically explore direct professional and educational links between UK-based Ayurvedic practitioners and Ayurvedic institutions or practitioners in India. Future research could examine how such transnational networks contribute to knowledge updating, professional development, and the adaptation of Ayurvedic practice within the UK context. Future research should include a broader range of stakeholders, including patients, policymakers, and Western medical practitioners, to further explore Ayurveda’s role within UK healthcare. The authors have also examined patient perspectives in a separate publication [54].

## 5. Conclusion

Although Ayurvedic practitioners face considerable challenges in the UK, this study highlights their role in navigating professional recognition and practising within a constrained healthcare system. Professional associations play an important role in sustaining and advancing Ayurvedic practice.

The findings indicate a need for research and policy initiatives that support the inclusion of less institutionally recognised CAM systems. Future work should explore pathways for equitable recognition, interdisciplinary collaboration, and integration within a more inclusive and responsive healthcare framework.

## Supporting information

S1 Table Summary of Themes and Subthemes with Illustrative Quotes. (DOCX)
