# Supplementary material for: Experiences and perspectives of Ayurvedic practitioners on non-communicable disease management in the UK: A qualitative study
Source: PLoS One. 2026 Oct 12;21(10):e0360754. doi: 10.1371/journal.pone.0360754 (PMC13657308; doi:10.1371/journal.pone.0360754)
Supplement: Supplementary file 1 [file pone.0360754.s001.docx]

**Summary of Themes and Subthemes with Illustrative Quotes**

| Theme and Subtheme | Summary | Illustrative Quotes |
| --- | --- | --- |
| 3.2.1 Role of Ayurvedic practitioners in NCD management 3.2.1.1 Negotiating the role of Ayurveda alongside Western medicine | Practitioners positioned Ayurveda in varied ways: some stressed its complementary role alongside biomedicine, while others framed it as a standalone system. These views reflect ongoing negotiations of Ayurveda’s place within a Western-dominated healthcare context. | *“We need to work hand in hand, not criticise each other, as there are areas in Western medicine that Ayurveda may be helpful for, such as NCD management.” (AP15)*  *“The Western medical system excels in areas like surgery, which is crucial. When a patient with breast cancer came to me, I advised her, ‘… you must have the surgery.’ She underwent surgery and chemotherapy, and we used Ayurveda to prevent recurrence.” (AP5)*  *“As a GP or surgeon, I would usually prescribe ibuprofen, Voltarol, or naproxen—anti-inflammatories these can have harsh adverse effects like ulcers. The role of Ayurveda is to provide a much safer alternative with natural herbal preparations. So I integrate it into my practice.” (AP6)*  *"I don’t say Ayurveda has a cure for all conditions; it has its limitations. At least for certain conditions where it does help, it can be complementary." (AP12)*  *“I think Ayurveda is one of the natural systems of health and wellness for prevention and health promotion. It stands as a mainstream alternative, with many people turning to such systems at some point in their lives.” (AP8)*  *“In India, Ayurveda is mainstream, whereas here it is treated as complementary. Sometimes people just don’t get much improvement with modern medicine, and there are lots of people who don’t want to take modern medicine too, so they look for natural methods like Ayurveda.” (AP3)* |
| 3.2.1 Ayurvedic practitioners’ role in NCD management 3.2.1.2 Patient-centred approach and empowerment | Ayurveda was described as holistic, individualised, and empowering, contrasting with Western medicine’s disease-centred approach. | *“In Ayurveda, we primarily see each person as an individual. It is a patient-centred approach rather than a disease-centred one … Even if two people had the same diagnosis, they would not necessarily receive the same treatment.” (AP3)*  *“I notice that people are very used to getting medication with quick effects … so they may have the same expectations with Ayurveda.” (AP2)*  *“A patient’s blood pressure medication was doubled without lifestyle advice. I prescribed herbs and recommended walking and diet changes—now she is off the medication.” (AP11)*  *"About 75% of my clients seek pain management for conditions like fibromyalgia, often relying on painkillers for temporary relief. For lasting results, they need to invest in dietary changes and lifestyle adjustments. With commitment, they could find a more permanent resolution to their pain." (AP 4)* |
| 3.2.2 Barriers to Ayurvedic practice in the United Kingdom  3.2.2.1 Structural barriers | UK Ayurvedic practitioners face unregulated practice, lack institutional support, accountability pressures, and absent standardised training. | *“As an Ayurvedic practitioner, I must be cautious about what I prescribe. If I prescribe something outside the pharmacopoeia … I will be responsible for that. Nobody is there to support me in case there is a problem.” (AP5)*  *“We are not a regulated profession; it would be good if it’s regulated because then only people who are fully qualified can practise.” (AP3)*  *“Anyone can practise Ayurvedic medicine in this country, it’s unregulated, and that is a barrier.” (AP6)*  *“Currently, there is no university that recognises the Ayurveda training programme … as long as these things are not there, it’s hard for practitioners to provide university-recognised training.” (AP1)*  *“We are not able to have basically support of an institution … Here we are on our own, so you have to be much more careful with the practice.” (AP3)*  *“MDU and GMC brought in restrictions … doctors with the MDU may not offer cover or provide protection if things went wrong.” (AP6)*  *“There isn’t really a blueprint for how people should deliver or do their practise… There is no standardised approach to how we practice.” (AP2)* |
|  | UK Ayurvedic practitioners adapt treatments creatively due to restricted herbs, mineral bans, high costs, and limited facilities. | *“In this country, fresh herbs for managing NCDs are not so readily available; if they are, it’s too expensive, and there are no Panchakarma facilities … so we must make do with what we have.” (AP17)*  *“In the UK, we are only allowed to use single herbs, and there’s a list of herbs we are allowed and a list of herbs we are not allowed, so we look at that first of all. Most associations ask the practitioners to look at that when they start to practice here." (AP1)*  *“Also in Europe and the UK we are not allowed to use minerals, … so we are only able to work with the herbs rather than other mineral preparations.” (AP3)* |
|  | Ayurvedic practitioners reported scarce referrals from Western doctors, citing scepticism, poor collaboration, and policy barriers. | *“The access to patients with NCDs is very limited here … it is difficult to get referrals.” (AP1)*  *“We treated a patient with rheumatoid arthritis who visited regularly over time. I coordinated openly with her rheumatologist, but such communication is rare because most Western practitioners are hesitant to refer patients to Ayurveda. It’s challenging to gain access to patients with NCDs without broader policy changes.” (AP6 )* |
| 3.2.2 Barriers to Ayurveda practice in the UK 3.2.2.2 Financial barriers | High costs, lack of NHS coverage, and strict import regulations limited affordability and sustainability of Ayurvedic practice. | *“In the NHS, NCD treatment is a bit free, whereas in other countries it’s not the same; with having the NHS free, you are competing with that for the patients.” (AP1)*  *“Availability and cost of Ayurvedic medicines is a major challenge, especially oils because they are very heavy to import and so it brings a higher cost.” (AP3)*  *“Since Ayurveda is not covered by the NHS, you must pay for it privately; thus many of the clients discontinue treatment because of the cost.” (AP 4)*  *“Making a living from it is a big challenge... I had to conduct other studies in psychology and do another job.” (AP5)  “The MHRA restricts the import of Ayurvedic medicines. They must be licensed here and submitted for research. Indian companies are not prepared to invest in the UK … because the returns are not there.” (AP6)*  *“Regarding testing, ideally, you want to be testing for heavy metals and pollutants in the herbs. In the UK, it’s a lot more expensive to do the testing … ideally every batch that comes from India should be tested, because they have different standards in India than here” (AP3)*  *“Because it’s not part of the health system, patients have to use out-of-pocket expenses, which sometimes some patients may not be able to afford, and that restricts their outcomes of their condition." (AP1)*  *“The financial factor … sometimes someone needs more treatment, but they might not be in a position to afford it, so you have to also reduce that.” (AP3)* |
| 3.2.3 Benefits of professional associations and networks 3.2.3.1 Professional guidance on legal and regulatory frameworks | Associations offered legal, ethical, and insurance support, helping practitioners work with more confidence. | *“Associations like the British Association of Accredited Ayurvedic Practitioners, or APA, once you are a member, you can get insurance from holistic insurance … that facilitates the practice of Ayurveda in this country.” (AP6)* |
| 3.2.3 Benefits of professional associations and networks 3.2.3.2 Continuous professional development support | Associations promoted CPD and peer learning, supporting best practices and professional development. | *“We have Ayurvedic professional associations, and they do CPD courses and things we can join to keep our practice up to date.” (AP5)*  *"I would say what has worked in terms of Ayurveda is now there are associations which monitor the practice of practitioners, which support the practitioners in terms of their continuous medical education.” (AP1)* |
| 3.2.3 Benefits of professional associations and networks 3.2.3.3 Evidence generation and research support | Associations encouraged scientific validation and collaborative research to strengthen Ayurveda’s credibility. | *“There is some research happening now … Integration requires much research, but without collaboration and research, we cannot bring it to policy.” (AP1)* |

*S1 Table 1****. Summary of Themes and Subthemes with Illustrative Quotes*** *This table summarises the key themes and subthemes identified in the study, highlighting practitioners’ roles in NCD management, barriers to practice, and the support provided by professional associations. Illustrative quotes exemplify participants’ perspectives and experiences for each theme.*
